# The Effect of Health Status on Urban Adaptation of the Rural Elderly after Migration

**DOI:** 10.3390/healthcare11121761

**Published:** 2023-06-15

**Authors:** Kangkang Wang, Min Li, Jie Lyu

**Affiliations:** 1 College of Economics and Management, Shenyang Agricultural University, No. 120 Dongling Road, Shenhe District, Shenyang 110866, China; 2019200179@stu.syau.edu.cn; 2 International Business College, Dalian Minzu University, No. 31 Jinshi Road, Jinshi Beach, Dalian Development District, Dalian 116650, China

**Keywords:** health, urban adaptation, rural elderly after migration, CHARLS

## Abstract

As urbanization accelerates in China, more and more rural elders are moving to cities to live with their children. However, they face challenges in overcoming cultural, social, and economic disparities and maintaining health in urban life, and health is important human capital that may have a significant impact on the urban adaptation of rural elderly migrants (REMs). Based on the 2018 China Health and Retirement Longitudinal Study (CHARLS), this paper constructs an indicator system to measure the level of urban adaptation of REMs. The health level and urban adaptation of REMs are studied in depth, and how to better help them adapt to urban life in order to provide a healthy living environment and a good lifestyle is explored. The empirical analysis finds the following: (1) good health helps REMs achieve a better level of urban adaptation. (2) REMs with good health status are more likely to go to community clubs for activities and do physical activities and thus improve their urban adaptation level. (3) There are significant differences in the effects of health status on urban adaptation among REMs with different characteristics. REMs with better health status in the central and western regions have significantly higher levels of urban adaptation than those in the eastern regions, and men have higher levels of urban adaptation than women. Therefore, the government should construct classification measures according to the differentiated characteristics of rural elderly migrants’ urban adaptation, and guide and support their stratified and orderly adaptation to urban society.

## 1. Introduction

Rural elderly migrants (REMs) in China are a special vulnerable group in the context of rapid aging, rapid urbanization and mobile China. REMs are those who have an agricultural household registration (hukou), have followed their children to live in the city for more than half a year, and have reached the age of 50 [1], with passive mobility as an important characteristic [2]; this is because, in China, intergenerational care is usually undertaken by the older members of the family, i.e., grandparents, not only because of the cultural tradition of intergenerational parenting in China, but also because older Chinese people are expected by their adult children to contribute to the development of their families in their later years. In this sense, the majority of older people in rural China are passively ‘involved’ in the process of family mobility, and thereby constitute a passive mobile population. China’s hukou system, established in the 1950s, designed to restrict internal population migration, and especially rural-to-urban migration, is a household registration system that determines an individual’s residence, welfare benefits, and access to public services. It classifies people into urban and rural hukou, leading to significant disparities. Urban hukou holders enjoy better social welfare, education, healthcare, and job opportunities, while rural hukou holders face restrictions and inequalities. The system influences employment, education, healthcare, and housing. In recent years, the Chinese government has initiated reforms to address these inequities and promote a more inclusive society. However, further efforts are needed to bridge the urban–rural divide and ensure equal opportunities for all Chinese citizens. In summary, the hukou system in China is a household registration system that classifies individuals into rural or urban categories, determining their access to various social services and benefits based on their registered location. The phenomenon of REMs originates from the urban–rural dichotomy in China, where the economic and social development of cities is more advanced than that of rural areas, and therefore cities have become an attractive place for farmers to migrate. The family planning policy, which was introduced in 1971 and formally written into the Chinese constitution in 1982, has led to the fact that rural elderly generally have only one or two children and, due to the household registration system, are not entitled to social security except for the meager pensions under the new rural insurance system; therefore, they have to rely on their children in their old age. Some of the rural elderly passively choose to move to the city with them. With the increasing aging of the population and the development of urbanization, the group of REMs will further expand. Data from the Chinese government website show that the size of China’s mobile population reached 390 million in 2021, while the total population aged 60 and above was over 260 million. The elderly migrant group and those who grow old in the place of migration become important constituent groups of the mobile population and the society on the move.

REMs generally face difficulties in urban adaptation [1], which becomes a negative influence on coordinated economic and social development. Because there are more job opportunities in cities, many young farmers move to cities in search of better employment opportunities and living conditions, and they usually bring their parents with them because respecting and caring for the elderly is a basic family responsibility and obligation in traditional Chinese culture. However, life in the city is not easy for the rural migrant elderly. Due to their lack of urban resident status, they often face restrictions and unequal treatment, such as not being able to enjoy the social welfare and medical coverage accessible to urban residents, and not being able to receive the preferential treatment of urban residents, such as subsidized healthcare services, better access to hospitals and medical facilities, and lower medical expenses compared to non-registered individuals or rural residents. REMs might encounter limitations in terms of medical insurance coverage, higher medical costs, or restrictions on receiving healthcare services at certain facilities, for instance, health records as a helpful instrument for supporting prevention and early detection of chronic diseases are not systematically carried out among elder rural migrants in their receiving city [3]. Since older mobile groups are often neglected, as they have mostly withdrawn from the formal labor market and are experiencing the last stage of their life course, they are often regarded as a group without special problems [4,5,6]. The change of living environment and space leads to migrants’ maladjustment to the environment [7]; “adaptation” is a lifelong endeavor, and REMs are no exception. In addition, due to their inability to adapt to urban life, their worsened health or longing for the countryside lifestyle and their relatives and friends, rural elderly who migrated with their children to the cities choose to return to the countryside of their own accord, becoming actively empty-nest elderly. The lack of daily care and spiritual comfort is the main cause of rural elderly suicide [8], which affects the coordinated economic and social development. In conclusion, the number of REMs is increasing in China and has become an important issue facing Chinese society. The government and the community need to work together to provide them with better services and support to ensure that they can enjoy the dignity and rights they deserve.

Health is an important influencing factor for REMs in their adaptation to urban areas. REMs face various challenges in the process of new urbanization, and one of the most important factors is health status, which is also a very basic human freedom [9]. The healthy migrant phenomenon has been observed in terms of self-rated physical health among migrants; namely, when migrants initially arrive in cities, they may exhibit a higher level of health compared to the local population [10], despite having a lower socioeconomic status. However, research studies have also indicated that rural–urban migrants are more susceptible to infectious diseases, posing significant health risks [11], and over time, this healthy immigrant effect is lost [12]. Therefore, it is important to analyze the mechanism and effect of health status on the urban adaptation of REMs in order to better develop urban security and policies for them. The effects of health status changes on the urban adaptation of REMs can be divided into three aspects: firstly, from the psychological point of view, the deterioration of the physical health of REMs will make them lack a normal psychological state [13] and affect their ability to learn new skills, acquire new information, and socially interact in the new environment; secondly, from the material point of view, the deterioration of the health of REMs will affect their ability to participate in urban life independently; third, socially, health can affect people’s expectations, longevity, strength, energy, and staying power [14]; if rural REMs experience deteriorating health conditions, their ability to contribute socially diminishes. In the event that they return to the countryside, they are unable to assist in caring for their grandchildren over multiple generations or aiding their children with household tasks. Consequently, they lose their value in terms of reciprocal support and are gradually perceived as a burden by their children.

Not many studies have been conducted on REMs in China, a special group arising from the dualistic structure of China, while most of the concerns about migrants are aimed at younger rural migrants, often known as “rural migrant workers who come to city for more money and better life”. There are very few studies that combine the triple attributes of rural household registration + mobility + old age and focus on this group in terms of policy care and academic concern. The current studies have mainly investigated the current situation of social adaptation and its influencing factors of REMs [15,16], but only briefly described the level of health status and influencing factors of REMs, while the research on the combination of urban adaptation and health of REMs is not systematic and in-depth, and the correlation between the two even less so. Therefore, in the current context of “rapid aging”, “mobile China”, and “new urbanization”, this paper investigates the impact of the health status of REMs from the perspective of urban adaptation. Does health status affect the urban adaptation of this special group of REMs? What are the mechanisms of influence? Are there any differences in the effects of health on the urban adaptation of REMs with different characteristics? The answers to the above questions provide a theoretical basis and policy reference for promoting population urbanization, facilitating the citizenship of REMs, and accelerating the integrated development of urban and rural areas.

## 2. Theoretical Analysis and Research Hypothesis

With the acceleration of urbanization in China, more and more elderly people in rural areas are moving to cities with their children, and this population movement brings challenges to REMs. In this context, it is important to explore the role of health status on the urban adaptation of REMs for the development of elderly health policies and urban planning. The health capital model provides a theoretical framework for exploring this issue. This paper will analyze the role of health status on the urban adaptation of REMs based on the health capital model. The adaptation status of REMs is not only a reflection of their own welfare level, but also determines the speed of their transition to citizenship.

The health capital model was proposed by Grossman in 1972 to explore the complex trade-offs in health management [17]. Grossman argued that health is a consumer good and a factor of production that can be used both as a consumer good to increase quality of life and improve well-being and as an input to generate productive time, and the additional productive time generated can be traded off between leisure time and work time to obtain greater utility. The model has a strong explanatory power to explain a variety of health phenomena such as the relationship between socioeconomic status and health. The role of health status cannot be ignored in the urban adaptation process. Higher levels of health imply more productive time producing health stock and producing urban adaptation.

First, health status has a direct impact on the urban adaptation of REMs. Physical health is the foundation for REMs’ adaptation to urban life. REMs with good health and physical fitness are better able to adapt to the pace and pressure of urban life. Urban living conditions are relatively better, and older adults have access to better medical services and health care resources. However, at the same time, the urban living environment may also have negative effects on the health of REMs, such as air pollution and irregular diet. Therefore, REMs need to maintain good health by following a healthy diet, enhancing physical exercise, and avoiding risky behaviors. On the contrary, REMs with poor health status may encounter more health-related problems during the urban adaptation process, which in turn affects their quality of life and urban adaptation. Studies have shown that health is an important factor influencing the willingness of mobile populations to stay [18]. Health status is closely related to economic adaptation and self-identity of the migrant population [19] and is also positively related to cultural integration [20]. Health status has a significant effect on the urban adaptation of dislocated farmers [21] and also affects the urban integration of accompanying parents [22]. Based on the above analysis, this paper proposes the following hypothesis:

**H1:**  *Health status affects the urban adaptation of REMs.*

Second, health status has an indirect effect on the urban adaptation of REMs. Health status can not only directly affect individuals’ urban adaptation, but can also improve their urban adaptation ability by improving their social interactions and social resource access [23]. The cognitive ability of older adults is positively correlated with social participation [24], and older adults with good health status are more able to actively participate in social activities [25], establish good social relationships, obtain more social resources and support, and promote their social integration [26], thus better adapting to urban life. Physical health status is strongly linked to health behaviors [27]. Health status can also moderate the relationship between critical thinking and health behaviors [28]. Conversely, REMs with poor health status may feel isolated and helpless during urban adaptation and have difficulties accessing effective social support, which may affect their urban adaptation ability, or, more seriously, compel them to return to their place of origin due to health problems, a phenomenon known as “salmon preference” [29]. Accordingly, the research hypothesis is proposed:

**H2:**  *REMs with good health are more likely to have stronger social connections in the urban environment.*

## 3. Research Data, Variables, and Methods

### 3.1. Data Sources

The data in this paper come from the China Health and Retirement Longitudinal Study (CHARLS) database. The survey is a large-scale interdisciplinary project hosted by the National Development Institute of Peking University and jointly implemented by the Social Science Survey Center of Peking University and the Peking University Youth League Committee. Using a scientific PPS sampling method, the household survey was conducted in 28 provinces (autonomous regions and municipalities directly under the central government) of mainland China among middle-aged and older households and individuals aged 45 or older, covering 150 counties and 450 villages (communities), and as of the 2018 follow-up, there were 12,400 households and about 19,000 respondents, which is a high-quality set of micro data with universal representation of Chinese residents that can be used for comprehensive and in-depth analysis of China’s aging problem and promote interdisciplinary research on aging in China. Because unified resident hukou cannot determine whether they are rural hukou or not, which affects the determination of the status of rural elderly people who move with them, a small number of unified resident hukou are excluded from this paper. The sample selection of this paper is based on the following criteria: from the 2018 data, this paper screened out the sample of elderly people with agricultural hukou, aged 50 years or older, who had lived in their children’s city for more than half a year, and after removing the sample with missing important variables, a final sample population of 790 individuals was used for this study.

### 3.2. Variables

#### 3.2.1. Explained Variable

Urban adaptation. H. Entzinger [30] argues that the social integration of international migrants should be examined in four dimensions: socio-economic, cultural, political, and acceptance or rejection of migrants by the subject society. Meanwhile, rural elderly migrants in China have their own special characteristics and show more adaptation to the city than social integration for the following reasons: on the one hand, China has a typical dualistic urban–rural structure with huge urban–rural differences, as well as rural elderly migrants. On the other hand, the motivation for the rural elderly to migrate is to take care of their offspring and to retire, and their economic sources mainly depend on their children’s support, and their non-personal labor income [1] and rural pensions are also very limited. Many REMs subsidize their families by working part-time and recycling waste and are financially dependent on their children. Therefore, this paper constructs a system of urban adaptation indicators for rural migrant elderly according to their characteristics from three aspects: urban life adaptation, social interaction adaptation, and psycho-cultural adaptation, as shown in Table 1.

Urban life adaptation refers to the fact that the REMs have to change their original living habits and concepts to adapt to “modern” urban life after entering the city. Therefore, this paper draws on existing research and selects ① Urban living conditions, which are divided into objective and subjective housing conditions. Many REMs living with their children live in industrial communities in the city; some of the housing is still relatively old, and the housing conditions are not ideal. The measurement of the objective condition of urban living is based on several factors. Firstly, the presence of rental housing indicates the existence of housing costs, which can negatively impact the adaptation of REMs by limiting their expenses in other areas. This is represented by assigning a value of 0 to renting a house and a value of 1 to no rental house. Additionally, the availability of certain amenities determines the objective living conditions. Factors such as the presence of electricity, running water, bathing facilities, separated bathroom, and natural gas are evaluated. Each affirmative response (“yes”) to these items contributes 1 point. Furthermore, the age of the housing where REMs reside is considered, with a higher value assigned to newer houses (ranging from 0 to 5). The subjective conditions of urban living are also taken into account. For instance, the indoor temperature of the house is assessed. If the temperature is deemed appropriate, 2 points are assigned. Colder or hotter temperatures receive 1 point, while very cold or very hot temperatures receive 0 points. Studies have indicated that better objective housing conditions correlate with higher subjective well-being among older individuals. Living in newer homes is associated with a greater subjective sense of well-being. On the other hand, older houses tend to have older infrastructure, potentially leading to poorer living conditions compared to newer houses [31]. ② Hygienic conditions. Living conditions and hygiene may indirectly reflect aspects of adaptation; adaptation is based on daily life and closely related to an individual’s subjective agency, and improved living conditions and hygiene signify the efforts of rural elderly migrants to adapt to urban life. The assessment made by the observer regarding the cleanliness of the indoor environment can serve as an indication of the overall quality of living conditions. When living conditions are favorable, the indoor environment is more likely to be tidy, whereas in contrast, a disordered indoor environment may suggest less favorable living conditions [32]. ③ Urban air quality perception, using the question “Are you satisfied with the air quality this year?” The options for the question are from extremely satisfied to not satisfied at all, with a score of 4, 3, 2, 1, and 0, respectively. Urban air quality is very different from rural air quality; urban air quality is based on urban industrial development, convenience of life, traffic, transportation, education, medical services, etc. REMs may not be satisfied with urban air quality because they have lived in the countryside for a long time, and higher satisfaction indicates that rural elderly people who have moved to the city are more likely to accept modern urban life. ④ Urban consumption level, in which the monthly household consumption amount is divided into 4 levels by community and assigned a score of 0–3. The level 0 indicates that the household consumption of REMs is at the lowest level, and the consumption level reflects to a certain extent the urban living level of REMs; the higher the household consumption level, the easier it is to adapt to urban life. The reason for choosing household consumption expenditure instead of household income level in this paper is that consumption is a better indicator of permanent household income than current income [33].

Social interaction adaptation measures the extent to which older people communicate, interact, and establish good relationships with others in the city, i.e., social interaction [34]. In this paper, with reference to related studies [35], the following questions were selected: ① Interpersonal interaction, expressed by the questionnaire “whether they visited friends in the past month” and “whether they provided help to relatives, friends, and neighbors who did not live together”, respectively, with 1 point for each activity attended. ② Community involvement, corresponding to the questionnaire “whether volunteering, dancing, etc.”, with 1 point for each activity participated. ③ Relationship with children, using the “satisfaction with children’s relationship” to measure the relationship with children, and the higher the satisfaction level, the higher the value assigned, with a score of 0–4. The children’s family is the first living place of REMs, and the way their children living in the city socially interact gradually tends to be the same as that of the city residents, so a proper relationship with children is an important indicator of the level of social interaction of REMs.

Psycho-cultural adaptation, i.e., the degree to which the elderly can adapt to different ideas, concepts, and various cultures, mainly reflects REMs’ adaptation to modern urban culture, changes in values, and the overall evaluation of their inner feelings towards urban life. In this paper, with reference to existing studies, we selected ① Digital cultural adaptation and found that Internet use was positively related to social adaptation [36], measured by “Do they use WeChat? Do you read your friends’ moments and make mobile payments?”, with 1 point for each item.② Learning ability, using the questionnaire “Do you learn new knowledge online? Do you manage money online? Do you use the Internet?”, with 1 point for each item. ③ Satisfaction with life. The higher the satisfaction, the higher the score, and the score is 0–4. ④ Language proficiency was measured by use of the local language, “What language did you use during the visit?” The use of local dialects was assigned a score of 2, Mandarin 1, and home language 0. Language level is an important reflection of cultural adaptation, and the use of the local language indicates that REMs have a good cultural adaptation. ⑤ Medical satisfaction, using the question “Are you satisfied with the quality, cost, and convenience of local medical services?”, and the higher the satisfaction level, the higher the value, with a score from 0 to 4. REMs are not only natural persons but also social persons, and their medical behavior and evaluation are not only influenced by biological and natural factors, but also closely related to the social environment. In fact, medical satisfaction is a psychological adaptation to the medical services of unfamiliar doctors in the urban society of strangers, which is a psychological transformation process, compared with the medical services of familiar doctors in the rural society of acquaintances.

In order to comprehensively evaluate the level of urban adaptation of REMs, this paper follows the methodology and steps of Zhang Chen et al. [37] to calculate an urban adaptation score using the entropy weight method (EWM). The weights of each indicator of urban adaptation are calculated to downscale the urban adaptation indicator system and provide the basis for subsequent descriptive statistics and econometric analysis. As an objective comprehensive evaluation method, the entropy weighting method determines the weights according to the variance information of the indicators, mines the information entropy of the indicator data, and uses the information entropy to calculate the weights of each indicator, which excludes the interference of human subjective factors and the bias brought by artificially set weights, and also circumvents the inherent defects of the factor analysis method in which the key factors are assigned to take the variance contribution rate lacking a theoretical basis. The method eliminates the influence of differences in the scale, order of magnitude, and nature of indicators, so as to correctly calculate the value of indicators, which is widely used in the field of social science.

After determining the weights of each indicator in the urban adaptation index system according to the entropy weighting method, the indicators are weighted and averaged based on the weights to obtain the comprehensive evaluation index of urban adaptation degree, with higher scores indicating a higher urban adaptation degree of REMs. At the same time, the same method is used to measure the scores of urban life adaptation, social interaction adaptation, and psycho-cultural adaptation. The composition and descriptive statistics of the urban adaptation index system of the rural migrant elderly in their place of migration are shown in Table 1.

#### 3.2.2. Explained Variable Descriptive Statistics

As shown in Table 2, the urban adaptation score for REMs was 0.2134, with REMs scoring lower and less adaptive. The mean score for adaptation to urban life was 0.6423, social interaction adaptation was 0.1056, which was the lowest among dimensions, consistent with Peng’s findings [2], and psycho-cultural adaptation was 0.1540. REMs generally had better adaptation to urban life, but lower adaptation to social interaction and psycho-cultural adaptation. REMs in the eastern region scored less than 0.001 higher than those in the central and western regions in terms of urban adaptation, and there was little difference in the level of urban adaptation between REMs in different economic regions. As shown in Table 3, individual characteristics of REMs also influenced their urban adaptation, with REMs with a high school education and above scoring an average of 0.2650 in urban adaptation, 33.02% higher than those with less than a high school education, indicating that REMs with higher levels of education have higher levels of urban adaptation. Males had a higher level of urban adaptation than females, and REMs with spouses moving in had a higher level of urban adaptation than REMs who entered the city alone.

#### 3.2.3. Explanatory Variable

Based on different definitions of health, investigators have used a variety of different ways to measure the true health status of respondents. In this paper, objective health is used as a measure of health based on the approach of the literature and the availability of primary data. The objective health indicator uses the instrumental activities of daily living (IADL) indicator [38], which measures the ability of REMs to perform social activities independently. The scores of six activities, such as doing housework, shopping, and managing money, were summed, with “unable to complete” assigned a value of 0, “have difficulty and need help” assigned a value of 1, and “have difficulty but can still complete” assigned a value of 2. The higher the value, the higher the objective health level. Self-rated health is a reasonable and accurate way to assess a person’s mental health indirectly by considering self-assessment. Empirical tests have also shown that self-rated health (Phealth) and objective health (IADL) have a significant positive correlation [39]. Phealth is classified into five levels based on the CHARLS questionnaire, which are “very good”, “good”, “fair”, “bad”, and “very bad” in order. In terms of specific settings, this paper further defines health = 1 as including “very good”, “good”, and “average”, and health = 0 as including “bad” and “very bad”.

#### 3.2.4. Mediating Variables

Community activities (Cclub) and moderate physical activities (Modpa) are the two mechanism variables in this article. Cclub was measured by whether REMs attended activities in the community activity room in the past month. Modpa was measured by whether REMs had undertaken moderate physical activity more than 10 min each time every week. Both community activities and moderate physical activities were treated as dichotomous variables, where “1” represents the presence of the activity and “0” indicates the absence of the activity.

#### 3.2.5. Control Variables

Based on existing studies [35,40], this paper selects richer and more comprehensive indicators affecting the urban adaptation of REMs at the individual, family, and regional levels as control variables. The control variables include four categories: ① social support variables available to REMs, log of pension income received by REMs, emotional support from children’s families (Vpcp, frequency of contacting their migrating parents), log of family financial support (Lnhsup, log of the amount of financial support received from their adult children (cash or in-kind payment)), amount of perceived social support (Pcarenum, when REMs feel that they need help, the number of people who can provide help when they need it), and the type of medical insurance (Medcaren, the sum of the number of various types of medical insurance in which REMs participate); ② individual characteristics of REMs, including gender, age, education, number of chronic diseases, work, and religious beliefs, etc.; ③ household characteristics of REMs, including log of financial assets (Lnfassets, cash, deposits, bond purchases), log of property income (Lnrent, rental land, house rent of homestead), spouse migration (Smig, if their spouse migrated with them), and number of adult children (Childnum); ④ regional characteristics variables and province dummy variable.

### 3.3. Statistical Model

To examine the relationship between health status and urban adaptation of REMs, we estimate the following equation:

(1) $$CAT_{i}=\alpha +\beta health_{i}+\eta \sum_{m=1}^{5}\;{\mathrm{SP}}_{i}+\delta {\mathrm{FC}}_{i}{+D}_{s}+\mu_{i}\;$$

where ${\mathrm{CAT}}_{i}$ is the explanatory variable, which refers to the standardized urban adaptation score of REMs. It is calculated using the entropy weighting method, and a higher value indicates better urban adaptation of REMs; ${health}_{i}$ donates health status of REMs; β is the key parameter to be estimated in this paper, and it measures the effect of physical health on the urban adaptation of REMs; ${SP}_{i}$ denotes the five types of social support available to REMs; ${\mathrm{FC}}_{i}$ denotes the family characteristics and individual characteristics of REMs; and $D_{s}$ denotes the province fixed effects.

## 4. Result

### 4.1. Descriptive Statistics

The characteristics of the variables we chose are shown in Table 4. It can be seen that the self-rated health value (Phealth) of REMs is good, 77.30%; the objective health score is 16.9; and the psychological health (CESD) score is 7.34, which is low; a higher score of the latter indicates more serious psychological problems, indicating that the overall health of REMs is relatively optimistic, which is consistent with Cui et al.’s [41] statistical results. The age distribution is mainly below 69 years old, accounting for 82.80%, among which 51.90% of REMs are below 60 years old, and from the age distribution, REMs belong to the low-age elderly. The education level of REMs is concentrated in junior high school or below, and the education level is low, and it can be said that they are almost uneducated. The proportion of REMs who migrated with their spouses (Smig) was 67.80%, the proportion of which was high, which was in line with the characteristics of the era of family-oriented migration. A total of 17.8% of REMs go to community club (Cclub) for activities. A total of 30.30% of REMs still need to work in the city to subsidize their families. It can also be seen that only 8.4% of the REMs have religious beliefs (Religion), which is a very low percentage. The proportion of those who do moderate physical activity more than 10 min each time every week (Mopa) is 47.97%, less than half.

In order to determine whether the characteristics of the REM population are either similar to or significantly different from the non-migrant elderly and make us believe the REM population are a quite different group, we conducted a comparison with the main characteristics among the rural elderly, the urban elderly, and REM through the grouping T-means test, and significant differences were found among the rural elderly, the urban elderly, and REM in terms of personal characteristics. Compared to the rural elderly, REM showed significantly better objective physical health, objective mental health, more perceived social support, a better education level, more financial assets, and likelihood of going to community activity rooms, while, when compared with the urban elderly, it was found that there were no significant differences in objective physical health between the two groups. REM had significantly poorer objective mental health and self-rated health compared to the urban elderly. The education level and financial assets of REM were also lower than those of the urban elderly. The probability of participating in community activity rooms was significantly lower for REM compared to the urban elderly. However, REM had a lower prevalence of chronic diseases compared to the urban elderly. The test results are not included in the body due to space constraints, so they are placed in the attachment Table A1 and Table A2.

### 4.2. Basic Regression Results

Prior to conducting the regression model for empirical analysis, a multicollinearity test and Pearson/Spearman correlation coefficient was performed to test whether there is multicollinearity among independent variables that could distort the model estimates. The results show that (see Table A3 and Table A4) the inflation factor VIF was less than 2, less than the critical value 10, and the absolute value of the correlation coefficient between variables was found to be less than 0.8, suggesting the absence of a substantial multicollinearity issue among the independent variables. Consequently, it is possible to construct a regression model [42] without significant concern for multicollinearity, supporting the conclusion that no significant multicollinearity existed among the independent variables. Due to space limitations, the test results, which indicate the absence of significant multicollinearity among the independent variables, have been included in an attachment table.

Table 5 reports the results of the baseline regressions, which have small coefficients because the dependent variables in this paper have values ranging from 0 to 1. Among them, column (1) controls only for the core explanatory variables, column (2) controls for the social support variables available to REMs, column (3) adds individual and family character variables, and column (4) adds further regional dummy variables. The results remained stable after gradually adding individual, household-level, and regional control variables, demonstrating the reliability of the model.

The urban adaptation effects of health status are all significant at the 1% significance level, which indicates that REMs with higher levels of health are more likely to achieve higher levels of urban adaptation. An increase of one unit of health increased the urban adaptation of REMs by 0.007, and the increased urban score accounted for 3.3% of the mean urban adaptation score (0.2134).

In terms of the social support received by REMs, the coefficient of the type of medical insurance was signified positive, indicating that the more comprehensive the type of medical insurance in which REMs participated, the higher the level of their urban adaptation. The economic support given by their children’s families had a significant positive effect on the urban adaptation of REMs, indicating that the economic support relaxes the labor time requirement for REMs and facilitates their interaction with urban residents, thus facilitating their urban adaptation. The amount of perceived social support had a significant positive effect on the urban adaptation of REMs, indicating that the social support felt by REMs gave them spiritual encouragement and psychological comfort when they faced the “unfamiliar” social environment in the city. The coefficient of children’s emotional support was negative but not statistically significant, indicating that children’s emotional support had no significant effect on urban adaptation. Regarding the individual characteristics of REMs, the coefficient of education level is significantly positive and significant at 1% significance level, indicating that higher education levels contribute to the urban adaptation of REMs. The age factor also has a significant effect on the urban adaptation of REMs: the older they are, the weaker the urban living ability, interaction ability, and adaptation ability, so the urban adaptation level decreases with incremental age, and aging is not conducive to the urban adaptation of REMs. The healthy aging and active aging measures are imperative. Household characteristics of REMs also have a significant impact, such as property income and financial asset income, with both contributing to urban adaptation of REMs.

### 4.3. Mediating Effect Test

The KHB model, which is currently used more frequently, was used for the intermediate effect test [43,44]. REMs in good health are more likely than unhealthy ones to go to community clubs for activities and engage in moderate-intensity physical activities [45,46], which are conducive to REMs’ exposure to urban residents and their understanding of urban customs and culture, as well as the lifestyles and behaviors of urban residents, while moderate-intensity physical activities, such as cycling, can expand the range of activities of REMs and facilitate their more comprehensively understanding the city and thus enhance their level of urban adaptation. As shown in Table 6, the community club variable and moderate-intensity physical activity accounted for 14.83% of the total effect of health status on improving urban adaptation among REMs. This indicates that going to community club and moderate physical exercise are indirect factors in promoting urban adaptation of REM. As shown in Table 7, the effect of going to community club is greater than that of moderate physical exercise. To enhance urban adaptation of REMs, it is important to focus on guiding them to actively engage in community clubs, as well as physical activities, thus facilitating interaction with local residents.

### 4.4. Robustness Analysis

#### 4.4.1. Oster Test

In terms of the exclusion of the problem of omitted variables, a natural question is whether the health of REMs varies based on other unobservable characteristics. That is, are there unobserved family and child characteristics, and do these characteristics affect the health of REMs as well as their urban adaptation? To verify that the results are not affected by omitted variables, the Oster [47] test for model omitted variables, which has been adopted by many in the mainstream economics literature [48,49], was performed.

Oster pointed out that there are three ways to perform the robustness test: (1) the range of δ values of the test. Given the value of $R_{\max}$ and calculating the value in the case of β = 0, the coefficients are generally considered to be stable when the value of δ is greater than or equal to 1. (2) The range of β values of the test. Given the values of $R_{\max}$ and δ, the range of β values is calculated. Then, the range of values is compared with 0. If the interval does not contain 0 values, the coefficient is stable. (3) The range of $R_{\max}$ values of the test. Given β = 0 and δ = 1, the values $R_{\max}$ are calculated to discuss the explanatory power of the unobservable variables. In practical applications, the literature mostly takes one approach to robustness testing. In this paper, referring to Zhang [50], the range of δ is tested to see if there is a possibility of missing variables, and if δ is greater than 1 or less than 0, the bias caused by unobservable variables can be considered small. As can be seen from Table 8, the ratio of Oster test δ is greater than 1 and the test passes, so the baseline regression results are robust.

#### 4.4.2. Replace Explanatory Variable and Model

To define health as an independent variable more specifically and purely, health was replaced with self-rated health (Phealth) and treated as a dichotomous variable and regressed again by means of a replacement model. The explanatory variables in the robustness test section are integrated urban adaptation, in which column (1) is an OLS regression, column (2) is a robust regression with M-estimation model, which further removes the effect of extreme values on the regression results, and column (3) is an IPWRA regression model, which is doubly robust [51]; that is, if either the treatment model or the outcome model is incorrectly specified but the other is correctly specified, then the estimates are still consistent. King and Nielsen [52] point out that IPWRA estimators are less prone to mis-matching on irrelevant observables. As shown in Table 9, the regression results are consistent with the baseline regression results in Table 6 and are significant at least at the 5% level of significance. The significant positive effect of self-rated health indicators (Phealth) on urban adaptation indicates that our results are robust to the fact that health status significantly and positively affects urban adaptation among REMs.

### 4.5. Heterogeneity Analysis

#### 4.5.1. Grouped by REMs’ and Regional Characteristics

There are large differences in REMs’ life course, education level, gender, region of migration, and the length of time in receiving cities, so regression in groups were conducted based on these characteristics, and a suest test was conducted on two groups of samples; the test results rejected the original hypothesis of no significant differences except for the age group and region group, that is, there were significant differences between different groups, as shown in Table 10a–d. First, we examined the differences in urban adaptation between the lower-aged REMs and the middle-aged and high-aged REMs by age group. After 60 years of age, the body functions decline significantly, and the body enters a comprehensive aging stage, and 60 years of age is the physiological turning point of quantitative to qualitative changes in the human body [53]; so, by using 60 years of age as a cut-off point, the regression found that the coefficients of the under-60 group were not significant, while the others were. Therefore, it can be concluded that the effect on urban adaptation of REMs’ health status in the over-60 group is better than the urban adaptation level of those under 60. Secondly, the previous descriptive statistics showed that the education level of REMs was generally lower; so, the education level of REMs was divided into two groups, junior high school or above and elementary school or below, and it was found that the health of REMs with high education levels was more significant in their urban adaptation promotion. Males received greater health urban adaptation promotion than females. To test the possible influence of regional differences in China on the urban adaptation of REMs, the in-migration areas were divided according to the Midwest and the East [54], and the estimated coefficients of health variables did not pass the significance test in the East, while the health coefficients were significantly positive in the Midwest at the 10% level of significance (*p* < 0.1). Therefore, the positive effect of health status on urban adaptation was greater for REMs in the Midwest than those in the East. As previous studies showed, migrant workers in the more affluent southeastern coastal cities feel less accepted, have greater social and economic externalities, and cultural and customary differences, and group differences in migration are latent mechanisms [55], the latter two of which may be the reason for REMs’ differences in areas, too. Finally, we conducted a sub-analysis of the REMs by grouping them into two groups according to their length of time in urban area: “in the analysis of grouped regression, it was found that the effect of health on urban adaptation is significant for samples of REMs who have migrated for a duration ranging from six months to five years”. However, among the samples of REMs who have migrated for over five years, the impact of health on urban adaptation becomes non-existent.

#### 4.5.2. Grouped by Urban Adaptation Dimensions of REMs

There are three dimensions of urban adaptation of REMs, namely, urban life adaptation, social interaction adaptation, and psycho-cultural adaptation. Previously, we only analyzed the impact of health status on the urban adaptation of REMs, but the impact on specific dimensions is unknown, so it is necessary to investigate the impact of health status on different dimensions and to determine whether there are differences in the impact. From the OLS regression results in Table 11, it can be seen that the health status coefficient is significantly positive, and the significance level is at least 5%. It shows that the health status has a significant role in promoting all dimensions of urban adaptation of REMs, and improving the health status of REMs is conducive to improving the all-around urban adaptation level of REMs.

## 5. Discussion

While there have been some studies focusing on the determinants of urban adaptation among REMs, few of them have considered the role of health status, let alone how health status influences the level of urban adaptation. This research paper aims to investigate the impact of health status on the urban adaptation of REMs in cities. Drawing on classical theories and the relevant literature, we propose hypotheses regarding the relationship between health status and the level of urban adaptation among REMs, and we utilize a national dataset from China to test these hypotheses. The primary objective of our study is to provide insights into the factors influencing the urban adaptation of REMs and to contribute to their integration into cities and the establishment of an inclusive society.

The findings of this study highlight the significance of REMs’ health status as a crucial factor in enhancing their urban adaptation. Our research has shed light on a previously understudied and less explored aspect of REMs’ urban adaptation. The results align with other studies that have focused on internal migrants’ settlement intentions and social integration in China, which have demonstrated the importance of physical health in influencing migrants’ intentions to permanently move to the city and their social integration, among other factors. When REMs experience poor health, their access to physical activities and community clubs may be restricted, impeding their interaction with the urban environment. Additionally, increased healthcare costs and difficulties in caring for their grandchildren may further exacerbate the challenges they face, potentially leading to their children viewing them as burdens. Consequently, REMs with a poor health status may exhibit lower levels of urban adaptation.

As previous studies have shown, the health of migrants has affected the social integration of migrants [19], and social participation, their length of urban residence, and social support from local residents can moderate the effect of health on migrants’ social integration level [56,57,58]. However, we point out that REMs who are also migrants but elderly ones and not in the formal labor market are more likely to go to community activity rooms for activities and to do moderate-intensity physical activities when they are in better health. In addition, in the robustness testing section, the Oster test and other methods have convinced us that health is a key factor in urban adaptation. This highlights the importance of health for REMs when urban adaptation is considered as the outcome.

Furthermore, the results of our study show that REMs with better physical health may have a higher urban adaptation level in terms of urban life adaptation, social interaction adaptation, as well as psycho-cultural adaptation. One of the most important findings is that REMs with different characteristics showed considerably different urban adaptation outcomes; thus, different measures for different characteristic groups constructed by the government are highly needed.

Our results have important theoretical implications for the study of internal elderly migrants from rural households because previous studies have generally ignored the health of elderly rural migrants when discussing factors affecting urban adaptation. In this study, physical health was discussed to provide a comprehensive understanding that is important not only for relevant research, but also for the government. Traditional theories and research usually consider health as an important factor influencing the willingness of internal migrants to settle but ignore the impact on the level of social integration, or consider the impact of health on migrants, but ignore the impact of health on the urban adaptation of special groups such as REMs. This study highlights the impact of health on the urban adaptation of REMs and elucidates the mechanisms of community clubs and physical activities in this process.

Concisely, in previous studies, institutional barriers, such as the hukou system and medical service, have been broadly discussed as key factors in migrants’ urban adaptation. This paper emphasizes the impact of an individual’s health on urban adaptation, which is more relevant and should not be overlooked when discussing elder migrants’ urban adaptation. As China’s urbanization advances, more rural elderly will move to urban areas to live with their children, and the Chinese government should implement targeted polices for protecting REMs’ health for family happiness, for inclusive urbanization, and for a harmonized society.

Due to the limitations of the data, there are some limitations in this paper. First, there are no specific tracking survey data for REMs, and the sample size selected from the CHARLS database is limited; thus, the analysis using cross-sectional data has difficulties dealing with endogeneity well on the one hand, and it cannot dynamically analyze the impact of time point changes on the other hand. The health status of REMs is a continuous and uncertain process, so the health of REMs cannot be judged only on the basis of a certain point in time. Second, this paper establishes a simple causal framework and attempts to explore how health affects the urban adaptation of REMs, but due to the lack of richer information (e.g., leisure time schedule, frequency and amount of contact with peer groups and urban residents, etc.), it is not possible to explore the influence mechanism in more depth. Finally, the reverse causality between community activity and physical activity and health status was also not addressed in this paper due to the lack of appropriate instrumental variables. The above limitations are the next step to try to alleviate or the problem to solve.

## 6. Conclusions

Based on data from the fourth China Health and Retirement Longitudinal Study in 2018, this study constructed urban adaptation indicators for REMs, measured urban adaptation scores for REMs, and portrayed the adaptation status of REMs. The OLS regression model, robust regression M estimation, IPWRA regression, and KHB mediated effects model were used to explore the effect of health status on urban adaptation of REMs. The basic conclusions drawn were the following:

First, according to the results of descriptive statistics and urban adaptation measures, REMs have a fair level of health, lower education, and a high proportion of spouses accompanying them. The level of urban adaptation was extremely low, and men had a slightly higher level of urban adaptation than women. REMs with high education levels and spouses accompanying them had a higher urban adaptation level.

Second, according to the results of the empirical analysis, the research hypotheses of this paper all held true. That is, health status does have a significant positive effect on the urban adaptation of REMs, and the mechanism analysis indicates that REMs with good health status have a higher possibility of going to community clubs for activities, engaging in physical activities, interacting with urban residents, expanding the scope of interaction, and thus promoting urban adaptation.

Third, there were significant differences in the effects of health status on urban adaptation among REMs with different characteristics. Health status had a more significant effect on urban adaptation among REMs in the central and western regions. The effect of increased urban adaptation level due to health of REMs with high education levels was greater than that of REMs with low education levels.

## 7. Policy Implications

The role of health status on the urban adaptation of REMs is multifaceted. It can not only directly affect urban adaptation of the elderly, but also influence their urban adaptation by affecting their community activities and physical activities. There are many factors affecting health, and different subjects play different roles in promoting the health of REMs. Therefore, in the process of urbanization, relevant subjects should work simultaneously to strengthen health protection and social support for REMs to promote their urban adaptation. First, children should care about the health of their parents who have moved with them. They can also make full use of green areas, parks, and community rooms in urban communities, so that the REMs can get in touch with the natural environment and the humanistic environment and gradually adapt to modern urban society. Since REMs are lonely and isolated, children should take the initiative to communicate with their migrating parents and provide psychological guidance to ease the discomfort of REMs due to the change of environment and cultural systems. Second, the community should establish a health monitoring and treatment system. The community is the “second environment” for REMs, and it is also the most important activity space. Thirdly, the city government can pay attention to the changes in the health status of the elderly and help them stay healthy in various ways, such as organizing public welfare activities and building health service platforms to help REMs adapt to urban life better. Finally, REMs should take the initiative to engage in physical and social activities, practice self-direction of negative emotions, and enhance physical and mental health.

## Figures and Tables

**Table 1:** Urban adaptation indicators for China’s rural elderly after migration.

Urban life adaptation	1. Urban living conditions 2. Hygienic conditions 3. Urban air quality perception 4. Urban consumption level	1. Availability of electricity, bathing facilities, toilet, running water, natural gas, whether renting [0–6]
		2. Age of housing occupied [0–5]
		3. Indoor temperature level [0–2]
		4. Indoor house tidiness [0–4]
		5. Air quality satisfaction [0–4]
		6. Level of monthly household consumption [0–3]
Social interaction adaptation	1. Interpersonal interaction 2. Community involvement 3. Relationship with children	1. Whether interacting with friends, providing help to friends, providing care to strangers [0–3]
		2. Whether involved in volunteering, dancing, etc. [0–2]
		3. Self-rated relationship with children [0–4]
Psycho-cultural adaptation	1. Digital cultural adaptation 2. Learning ability 3. Life satisfaction 4. Language proficiency 5. Medical satisfaction	1. Whether using WeChat, friends circle, and mobile payment [0–3]
		2. Whether surfing the Internet, learning financial knowledge, and taking online courses [0–3]
		3. Life satisfaction [0–4]
		4. Language ability [0–2]
		5. Medical satisfaction with local hospitals [0–4]

Note: In order to eliminate the influence of the dimension, the urban adaptation index of rural elderly migrants is standardized to be in the interval of [0, 1], and the larger the value, the higher the level of urban adaptation of the dimension.

**Table 2:** Urban adaptation status of REMs.

Urban Adaptation Dimension	Total Sample (n = 790)		East Region (n = 272)		Mid-West (n = 518)	
	Mean	S.D.	Mean	S.D.	Mean	S.D.
Urban adaptation	0.2134	0.1688	0.2138	0.1710	0.2132	0.1679
Urban life adaptation	0.6423	0.2071	0.6331	0.2201	0.6471	0.2001
Social interaction adaptation	0.1056	0.1521	0.1081	0.1493	0.1043	0.1537
Psycho-cultural adaptation	0.1540	0.2382	0.1504	0.2317	0.1558	0.2418

**Table 3:** T-means test for urban adaptation among REMs of different characteristics.

Variables	N1	Mean1	N2	Mean2	Mean_Diff
Education (Low)	466	0.1775	324	0.2650	−0.0875 ***
Smig (Not migrated)	254	0.1813	536	0.2286	−0.0473 ***
Gender (Female)	405	0.1993	385	0.2282	−0.0289 **

Note: ** *p* < 0.05, *** *p* < 0.01.

**Table 4:** Descriptive statistics.

Variables	Description	Frequency	Percentage
Gender	Female	405	51.3
	Male	385	48.7
Age	50–59	410	51.9
	60–69	244	30.9
	70–79	93	11.8
	80–	43	5.4
Education	No degree	39	4.16
	Primary school	415	44.29
	Middle school	409	43.65
	High school and above	69	7.89
Modpa	No	411	52.03
	Do moderate physical activity more than 10 min each time every week	379	47.97
Smig	No	254	32.2
	Migrated with their spouses	536	67.8
Cclub	No	649	82.2
	Go to community activity club for activities	141	17.8
Work	No	551	69.7
	Ever worked in the city	239	30.3
Phealth	Poor	179	22.7
	Good (according to self-rated health question in questionnaire)	611	77.3
Religion	No	724	91.6
	Have any religious belief	66	8.4
Continuous variables	Description	Mean	(S.D)
Lntranspen	Log of yearly pension	2.96	3.87
Pcarenum	Perceived social support (the number of people who can help them when they need help in the future)	1.08	0.86
Medcaren	Number of medical insurance types in which they participated (such as urban and rural resident medical insurance, private medical insurance)	1.04	0.3
Vpcp	Emotional support from children (the frequency of children visiting and contacting their migrant parents)	8.49	4.7
Lnhsup	Log of the amount of financial support received from their adult children (cash or in-kind payment)	6.2	3.59
Childnum	Number of adult children	2.47	1.13
IADL	Objective health score (calculated from questionnaire items)	16.91	3.05
CESD	Psychological health score (calculated from questionnaire items)	7.34	6.23
Lnrent	Log of property income (rent of leased land and homestead house)	1.76	3.16
Lnfassets	Log of income from financial assets (cash, deposits, and bond)	8.72	1.8
Disnum	The number of chronic diseases they acquired	2.16	1.95

**Table 5:** Empirical test results of benchmark regression.

	(1)	(2)	(3)	(4)
IADL	0.0113 *** (0.0009)	0.0096 *** (0.0009)	0.0057 *** (0.0012)	0.0070 *** (0.0013)
Medcaren		0.0544 ** (0.0227)	0.0391 * (0.0224)	0.0404 * (0.0221)
Lnhsup		0.0020 (0.0017)	0.0038 ** (0.0017)	0.0035 * (0.0018)
Pcarenum	.	0.0214 *** (0.0075)	0.0160 ** (0.0077)	0.0175 ** (0.0079)
Vpcp		−0.0004 (0.0014)	−0.0011 (0.0015)	−0.0011 (0.0015)
Lntranspen		−0.0048 *** (0.0015)	−0.0002 (0.0018)	−0.0006 (0.0019)
Education			0.0069 *** (0.0014)	0.0078 *** (0.0015)
Smig			−0.0011 (0.0129)	−0.0071 (0.0128)
Gender			−0.0036 (0.0134)	−0.0069 (0.0140)
Disnum			0.0028 (0.0033)	0.0045 (0.0035)
Age group (ref. 50–59)				
60–69			−0.0457 *** (0.0159)	−0.0404 ** (0.0164)
70–79			−0.0598 *** (0.0202)	−0.0569 *** (0.0208)
80 and above			−0.0564 *** (0.0206)	−0.0604 *** (0.0225)
Religion			0.0123 (0.0189)	0.0163 (0.0193)
Work			−0.0106 (0.0145)	−0.0210 (0.0146)
Lnfassets			0.0079 ** (0.0036)	0.0079 ** (0.0035)
Lnrent			0.0044 ** (0.0019)	0.0035 * (0.0019)
Childnum			−0.0032 (0.0052)	−0.0024 (0.0054)
Province-fixed	No	No	No	Fixed
Observations	790	790	790	790
R^2^	0.0415	0.0779	0.1480	0.1849

Note: * *p* < 0.1, ** *p* < 0.05, *** *p* < 0.01. The parentheses are reported as robust standard errors.

**Table 6:** Summary of direct effect and indirect effect.

Effect	Coef.	Z	*p* > z	Proportion
Reduced (Total Effect)	0.0067 ***	5.7562	0.0000	100%
Full (Direct Effect)	0.0057 ***	4.7499	0.0000	85.17%
Diff (Indirect Effect)	0.0010 ***	2.7418	0.0060	14.83%

Note: *** *p* < 0.01.

**Table 7:** Disentangled contributions of mediators.

Intermediate Variable	Coef.	Standard Error	Indirect Effect Prohibition	Total Effect Prohibition
Community club	0.0005	0.0003	54.77%	8.12%
Physical activity	0.0005	0.0003	45.23%	6.71%

**Table 8:** Results of the Oster test.

Dependent Variable	Controls in the Restricted Set	Controls in the Full Set	Oster Test (δ)
Urban adaptation	Controls selected in outcome equation	All potential controls	1.3189

Notes: Cell in the last column reports the ratio calculated by the method proposed by Oster (2019). We assume that R^2^, with unobservable and observed variables included, is 1.3 times the R^2^ of the regression with a full set of controls, as suggested by Oster (2019). Province-fixed effects are included in both restricted and unrestricted estimations.

**Table 9:** Robust test: replace key exploratory variable and models.

Variables	(1)	(2)	(3)
Phealth	0.0377 *** (0.0135)	0.0235 ** (0.0101)	0.0410 *** (0.0132)
Other control variables	Yes	Yes	Yes
Province-fixed effects	Fixed	Fixed	Fixed
Observations	790	790	790
R^2^ (pseudo R^2^)	0.1823	0.0860	-

Note: ** *p* < 0.05, *** *p* < 0.01. The parentheses are reported as robust standard errors. Column (3) shows the ATE coefficient.

**Table 10:** (**a**) Heterogeneity analysis grouped by age. (**b**) Heterogeneity analysis grouped by education. (**c**) Heterogeneity analysis grouped by gender. (**d**) Heterogeneity analysis grouped by region. (**e**) Heterogeneity analysis grouped by length of time in urban area.

(a)			
	(1)	(2)	(3)
	Ungrouped	Below 60 years old	60 + years old
IADL	0.0070 *** (0.0023)	0.0084 (0.0087)	0.0082 *** (0.0019)
Other control variables	Yes	Yes	Yes
Province-fixed effects	Fixed	Fixed	Fixed
Observations	790	410	380
R2	0.1756	0.1621	0.2311
**(b)**			
	**(1)**	**(2)**	**(3)**
	Ungrouped	Primary school or below	Junior high school or above
IADL	0.0070 *** (0.0023)	0.0051 ** (0.0021)	0.0112 * (0.0065)
Other control variables	Yes	Yes	Yes
Province-fixed effects	Fixed	Fixed	Fixed
Observations	790	261	529
R^2^	0.1400	0.1358	0.1009
**(c)**			
	**(1)**	**(2)**	**(3)**
	Ungrouped	Female	Male
IADL	0.0070 *** (0.0023)	0.0065 *** (0.0023)	0.0149 *** (0.0051)
Other control variables	Yes	Yes	Yes
Province-fixed effects	Fixed	Fixed	Fixed
Observations	790	405	385
R^2^	0.1756	0.2501	0.2195
**(d)**			
	**(1)**	**(2)**	**(3)**
	Ungrouped	East	Midwest
IADL	0.0070 *** (0.0023)	0.0058 (0.0035)	0.0058 * (0.0031)
Other control variables	Yes	Yes	Yes
Observations	790	272	518
R^2^	0.1372	0.1740	0.1403
**(e)**			
	**(1)**	**(2)**	**(3)**
	Ungrouped	Less than 5 Years	More than 5 Years
IADL	0.0080 *** (0.0021)	0.0075 *** (0.0023)	0.0098 (0.0079)
Other control variables	Yes	Yes	Yes
Province-fixed effects	Fixed	Fixed	Fixed
Observations	790	690	100
R^2^	0.1770	0.1974	0.3892

Note: * *p* < 0.1, ** *p* < 0.05, *** *p* < 0.01. The parentheses are reported as robust standard errors.

**Table 11:** Heterogeneity analysis grouped by dimension of urban adaptation.

	(1)	(2)	(3)	(4)
	Urban Adaptation	Urban Life Adaptation	Social Interaction Adaptation	Psycho-cultural Adaptation
IADL	0.0070 *** (0.0013)	0.0076 ** (0.0036)	0.0061 *** (0.0014)	0.0043 ** (0.0017)
Other control variables	Yes	Yes	Yes	Yes
Province-fixed effects	Fixed	Fixed	Fixed	Fixed
Observations	790	790	790	790
R2	0.1849	0.1346	0.1266	0.1603

Note: ** *p* < 0.05, *** *p* < 0.01. The parentheses are reported as robust standard error.

## Data Availability

The data that are presented in this study are available on request from the corresponding author. The data are not publicly available due to privacy restrictions.

## Appendix A

**Table A1:** Grouping T-means test between rural elderly and REM.

	N	N1	N2	Mean1	Mean2	Mean_Diff
IADL	10,494	9704	790	16.4000	16.9101	−0.5101 ***
CESD	10,374	9584	790	7.8122	7.3430	0.4691 **
Phealth	9701	8911	790	0.7217	0.7734	−0.0517 ***
Pcarenum	10,494	9704	790	1.0262	1.0810	−0.0548 *
Education	10,494	9704	790	3.9697	5.6848	−1.7151 ***
Lnfassets	10,494	9704	790	8.2380	8.7241	−0.4861 ***
Disnum	10,494	9704	790	2.0986	2.1633	−0.0647
Cclub	10,492	9702	790	0.1490	0.1785	−0.0294 **

* *p* < 0.10, ** *p* < 0.05, *** *p* < 0.01.

**Table A2:** Grouping T-means test between urban elderly and REM.

	N	N1	N2	Mean1	Mean2	Mean_Diff
IADL	3585	2795	790	17.0132	16.9101	0.1031
CESD	3569	2779	790	5.4005	7.3430	−1.9425 ***
Phealth	3317	2527	790	0.8322	0.7734	0.0588 ***
Pcarenum	3585	2795	790	1.0318	1.0810	−0.0492
Education	3585	2795	790	8.6447	5.6848	2.9599 ***
Lnfassets	3585	2795	790	9.1949	8.7241	0.4707 ***
Disnum	3585	2795	790	2.4386	2.1633	0.2753 ***
Cclub	3584	2794	790	0.2462	0.1785	0.0678 ***

*** *p* < 0.01.

**Table A3:** Variance inflation factor of each variable.

	VIF	1/VIF
IADL	1.558	0.642
Lnfassets	1.103	0.907
Lnrent	1.037	0.964
Childnum	1.543	0.648
Education	1.279	0.782
Smig	1.269	0.788
Gender	1.361	0.735
Disnum	1.131	0.884
2.agegroup	1.88	0.532
3.agegroup	1.986	0.504
4.agegroup	1.877	0.533
Religion	1.062	0.941
Work	1.258	0.795
Medcaren	1.063	0.94
Lnhsup	1.305	0.766
Pcarenum	1.095	0.913
Vpcp	1.264	0.791
Lntranspen	1.819	0.55
Mean VIF	1.383	

**Table A4:** Pearson/Spearman correlation coefficient of each variable.

Var	1	2	3	4	5	6	7	8	9	10	11	12	13	14	15	16	17
Urban Adaptation	1.00	0.23	0.14	0.07	0.14	0.05	−0.10	0.24	0.18	0.08	−0.05	−0.23	−0.07	0.05	0.19	0.15	−0.12
IADL	0.20	1.00	0.12	−0.04	0.18	0.11	−0.13	0.27	0.26	0.20	−0.29	−0.32	−0.10	0.21	0.23	0.02	−0.20
Medcaren	0.13	0.08	1.00	0.06	0.13	0.08	−0.06	0.08	0.11	0.01	−0.03	−0.14	−0.09	0.07	0.09	0.10	−0.04
Lnhsup	0.03	−0.06	0.07	1.00	0.03	0.06	0.15	−0.04	0.01	0.05	0.11	0.25	0.02	−0.10	−0.03	0.01	0.33
Pcarenum	0.14	0.08	0.13	0.05	1.00	−0.02	0.03	0.09	0.21	0.13	−0.08	−0.06	−0.03	0.11	0.12	0.08	−0.04
Vpcp	0.05	0.15	0.06	0.21	−0.01	1.00	−0.20	0.13	0.08	0.00	−0.04	−0.23	−0.04	0.13	0.00	0.01	−0.30
Lntranspen	−0.13	−0.15	−0.05	0.15	0.03	−0.18	1.00	−0.16	−0.08	−0.06	0.14	0.62	0.00	−0.23	−0.04	−0.04	0.21
Education	0.27	0.25	0.07	−0.06	0.10	0.10	−0.17	1.00	0.22	0.29	−0.11	−0.32	−0.13	0.19	0.24	0.10	−0.18
Smig	0.13	0.29	0.09	0.01	0.21	0.08	−0.09	0.22	1.00	0.29	−0.04	−0.23	−0.09	0.08	0.19	0.06	−0.12
Gender	0.09	0.18	0.02	0.06	0.14	0.00	−0.06	0.30	0.29	1.00	−0.11	−0.04	−0.15	0.31	0.21	0.04	−0.05
Disnum	−0.05	−0.26	−0.03	0.12	−0.08	−0.02	0.14	−0.10	−0.05	−0.09	1.00	0.20	0.04	−0.16	−0.15	0.02	0.07
Agegroup	−0.24	−0.45	−0.12	0.24	−0.07	−0.22	0.56	−0.34	−0.27	−0.07	0.17	1.00	0.13	−0.29	−0.18	−0.05	0.44
Religion	−0.03	−0.07	−0.09	0.03	−0.04	−0.03	0.02	−0.13	−0.09	−0.15	0.02	0.12	1.00	−0.06	−0.01	0.03	0.03
Work	0.08	0.18	0.08	−0.09	0.12	0.10	−0.24	0.20	0.08	0.31	−0.15	−0.29	−0.06	1.00	0.17	0.03	−0.20
Lnfassets	0.16	0.18	0.07	−0.03	0.11	−0.01	−0.01	0.18	0.14	0.16	−0.14	−0.14	−0.04	0.12	1.00	0.09	−0.15
Lnrent	0.14	0.03	0.10	0.03	0.08	0.01	−0.04	0.10	0.07	0.04	0.02	−0.05	0.03	0.03	0.08	1.00	−0.05
Childnum	−0.13	−0.27	−0.04	0.29	−0.06	−0.27	0.23	−0.21	−0.17	−0.07	0.10	0.49	0.03	−0.21	−0.12	−0.06	1.00
